# Universities as Partners in Primary Health Care Innovation

**DOI:** 10.3389/fpubh.2021.713177

**Published:** 2021-08-10

**Authors:** Henryk Majewski

**Affiliations:** ^1^Access Health and Community, Richmond, VIC, Australia; ^2^Centre for Mental Health, Swinburne University of Technology, Hawthorn, VIC, Australia

**Keywords:** university, primary care, partnership, innovation, community

## Abstract

Universities have a unique role in the health ecosystem as providers of trained staff and discoverers of health innovations. However, often they sit in silos waiting for their rare blockbuster discoveries to change clinical care or seeing health services simply as future employers of their graduates or clinical trial sites. It is a transactional and targetted relationship. This present case study is of a primary health service Access Health and Community (AccessHC) in Australia and its university partner Swinburne University of Technology. Together they established a Kickstart Program which was to provide seed funding for small joint innovation projects generated by both organisations. One project exemplifies the approach. Swinburne who has a Design School was encouraged through the Kickstart Program to design a clinical waiting room of the future. This project started with a needs analysis. The written report was to inform the design. University staff linked with their internal University animations expertise to better communicate the needs analysis. The “Access me Not” animation was created, unknown to the staff at AccessHC. At initial presentation, the way the animation communicated was not imaginable by AccessHC. “Access me not” was submitted for the 2018 International Design Awards and received an honourable mention. However, the AccessHC staff saw other uses for the approach and contacted Swinburne to design a client journey animation for the newly introduced National Disability Scheme (NDIS). The co design produced an animation of immense help to parents in navigating the scheme for complex and chronic disability care and for AccessHC the scripting served as a framework to develop it new internal NDIS care systems and processes. The Swinburne team is now producing health navigation animations for the State Department of Health and Human Services. The Kickstart Program was an engagement strategy that has produced a set of health communication tools that the health service could not have envisaged and which the University could not have imagined an application. Small low risk seed funding can indeed introduce innovations and create beneficial relationships between health services and universities.

## Introduction

Universities occupy a privileged place in society as centres of the creation of knowledge and core facilitators of advanced teaching. Universities are significant economic entities within the communities in which they are located. There has been historical research into the relationship between Universities and the communities in which they are embedded (1). However, University linkages to communities in which they are located are often weak and there has been limited research into how to improve the connexions between Universities and communities, including commercial entities (2, 3). The focus has been on linkages with large corporations rather than community organisations which are often excluded in the analyses. There may be multiple reasons for the perceived lack of interaction between Universities and primary care organisations.

Firstly, in health care, the tertiary sector is well-organised in large hospital networks in most countries and is well-understood. Primary health care, on the other hand, involves the local diagnosis and treatment of acute and chronic illnesses in health care settings outside of hospitals. This also includes health promotion and disease prevention programs. Primary health care is often in the hands of small and even solo scale practises. In 2018, only 15 of the 38 countries in the OECD had primary health care services based on integrated teams or networks (4). This fragmentation makes interactions and translation of research between Universities and primary health difficult because of different scale, different resource capabilities and limited opportunities for cross communication. Small community organisations do not have the ability to expend resources in developing the relationship (5). In a review of primary health care research in Canada it was found that there was limited capacity within the primary health care sector for research (6). Partly this was due to the service delivery imperatives but also the isolation of potential researchers within small organisations. This is similar to many countries.

The University drivers for engaging in primary health research and innovation are not strong, especially outside the discipline of general medical practise.

In general, most governments fund primary health research at a lower level than other areas of health research as for example happens in the UK (7), despite the UK being an example of a well-organised primary health system. Major primary health journals are lowly rated in the health journal rankings (see^1^). There is also a bias towards traditional research areas which are better funded and where the risks and rewards are well-understood. An impact at a local community level may be perceived to be if less value than broader research areas, especially those that are product and not systems oriented. Finally, community based innovation is unlikely to produce a commercial pay off so commercially funded research in the sector is also limited.

The initiation of this case study was the dilemma of a resource constrained, primary health service Access Health and Community (AccessHC), with little tradition of innovation. AccessHC sought to embed an innovation culture to support service development and improvement. The task was set as a whole of organisation initiative applicable to all health and clinical disciplines within its services. AccessHC reached out to Swinburne University of Technology (Swinburne) in Melbourne, Australia. with a view to develop a sustainable innovation partnership which was of mutual benefit. From the outset the case recognised that the drivers for the University to participate in the collaboration were not strong and the emphasis was on relationship development rather than a targeted research area.

The case describes the processes which led to the establishment of an ongoing relationship and some of the tangible outcomes for both parties in primary health care.

## Context

Melbourne, Australia is a large well-developed city of about 5 million residents with sophisticated Universities, health research institutes and teaching hospitals. Its population has access to free health care in a public/private model but disparities in health access for people of different economic status are significant. Access Health and Community is a small not for profit, independent primary health service in Melbourne Australia. It provides access to health services regardless of ability to pay. It is a charity. Relative to hospital networks in Melbourne, AccessHC is small and relies on a mix of government and fee for service income. It is different from solo-practise primary health care in that it sees its role as integrative and delivers almost all primary care disciplines and activities with over 300 staff. The clinical delivery areas are broad leading to an aspirational multidisciplinary approach over primary care medicine, mental health, dentistry, aged care health services, disability care, nursing, and physical therapy. Its purpose is “Building Healthier Lives Together” [See (8)]. In terms of primary health services, it fits the description by the OECD of being part of a typically small and fragmented system (4). Innovation and change are at the edge of possibilities for AccessHC in a budget stretched to meet community demand.

Swinburne University of Technology has about 42,000 students and 3,000 staff in Melbourne. It is one of seven Universities in Melbourne, Australia. Its footprint in health is small relative to some of the other universities in Melbourne. It does not have either a medical or dental school for example and at the start of the case study no nursing and limited allied health programs. Part of its strategic plan is to connect with business, industry, and community (9). It only recently became a University in 1992.

Prior to 2014, the two organisations had little contact beyond some linkages of individual staff members. The main Swinburne Campus was less than 15 metres from the largest AccessHC clinic. The question was could the two organisations connect for mutual benefit. The case began in 2014 and was still ongoing in 2021.

An important part of the context was the limited direct disciplinary connexion between Swinburne University and AccessHC. This prompted a focus on relationship and cross-disciplinary relationships rather than a targeted research topic.

## Detail

### Starting the Relationship

The initial steps were from AccessHC in a reach out for a collaboration with Swinburne. Initially this included information about AccessHC for distribution through the Swinburne University Office of Collaborations and Partnerships (10). This office proved fundamental in creating opportunities with the wider university beyond expected health faculty contacts in all phases of the program. Indeed, all areas of the University engaged in discussions including more distant disciplines such as accounting and engineering. Looking back, the most prolific engagements and outcomes came from the Design faculty, not health.

**Figure 1:**
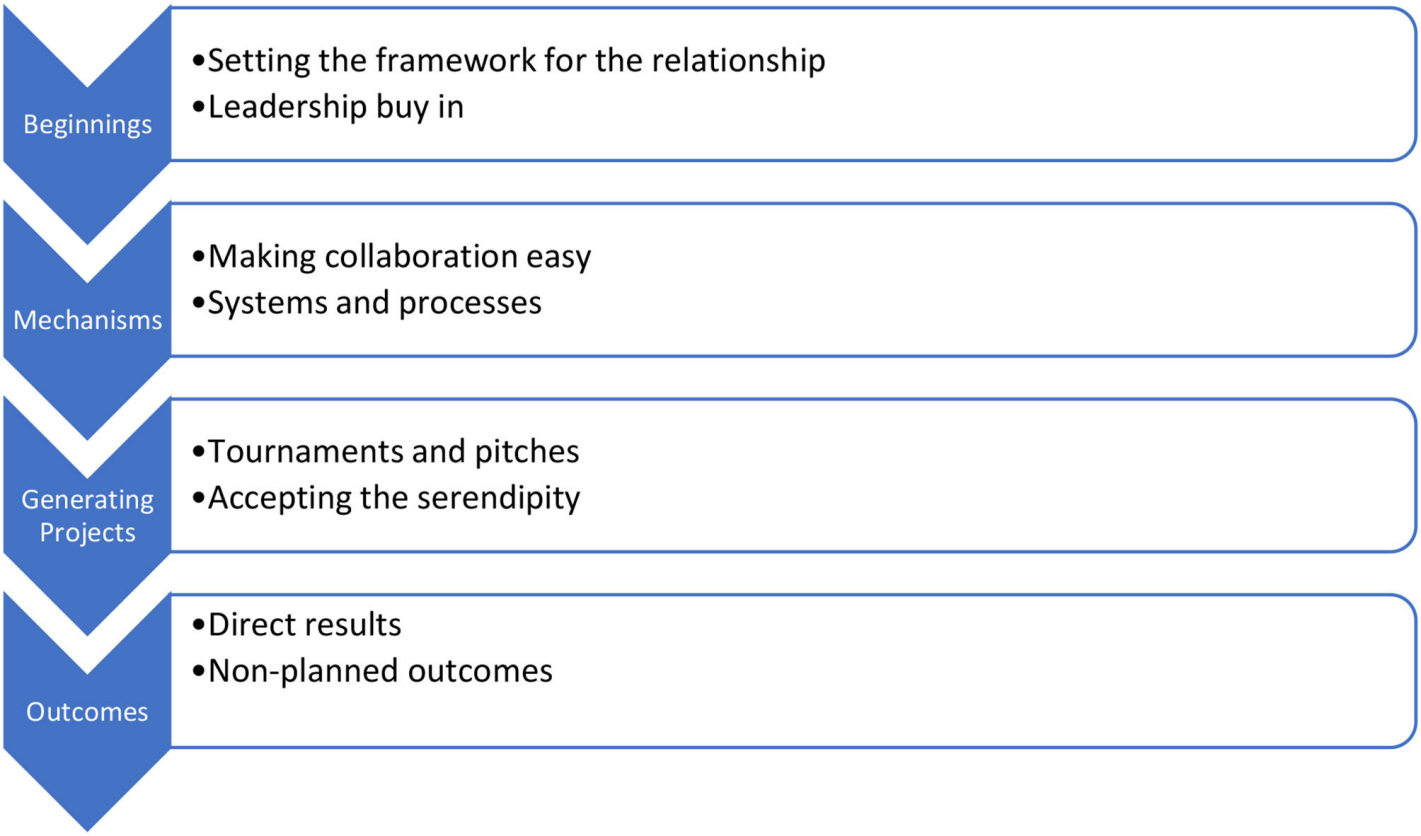
Steps in the University Community relationship case.

As can be seen in Figure 1 the initial step was setting the framework for collaboration and leadership buy in. A memorandum of understanding (MOU) was created. This was a non-legally binding document that described both organisations desire to collaborate. This was signed off at the highest levels of both organisations: the Vice Chancellor in the case of Swinburne University and the Chair of the Board in the case of AccessHC [See (11)]. The high-level support was fundamental in establishing the collaboration as important for both organisations through public acknowledgment to both staff groups. The MOU was signed after about 18 months of low-level interaction. For AccessHC, it was a visible step in making innovation important for the strategic leadership group which largely was focused on operational development. The MOU gave a prestige touch point for the Board of Directors of AccessHC to value the relationship and commit to its success despite important competing demands of a small health service.

Behind the memorandum of understanding were two other initiatives. The first was the Kickstart Program. The Kickstart Program is a fund created by AccessHC to facilitate collaborations with Swinburne. Its intention was small scale seed funding to facilitate interactions between AccessHC and Swinburne University and to create links between Swinburne and AccessHC. The Kickstart Committee had equal numbers of representatives from each organisation. Its annual budget was modest between $30,000 and $50,000 (AUD). The preamble was: “AccessHC has the ambition to be an excellent primary health service founded on encouraging innovation. In these Kickstart Fund Projects, AccessHC is looking for ideas about something that ultimately would make a practical difference. Some ideas will come from AccessHC but equally ideas may come from Swinburne.” It should be noted that from the outset there was not an overarching project idea, discipline area or single focus. Rather the Kickstart Program was designed to facilitate the relationship. To some extent the Kickstart Program was an incentive for Swinburne University to value to relationship, at least in the beginning. It also was a process which both organisations could contribute to meaningfully.

The projects funded by the Kickstart Program were governed by a Master Research Agreement between Swinburne and AccessHC and this was the second background structure. This agreement detailed generic legal project requirements including intellectual property arrangements. Its chief benefit was that any new project could be approved quickly without reference to legal representatives of either organisation or any new negotiation. The agreement protected existing intellectual property but gave AccessHC certain rights on any intellectual property created by the project. This was the second important step in Figure 1 in having the systems and processes to make collaboration easy.

### Generating the Ideas: Innovation Tournaments

From the outset, AccessHC had only a small understanding of the interests and capabilities of Swinburne University to contribute to its health service. AccessHC also did not have a firm idea of its own problems seeking solutions. There was an ever present operational focus which was all consuming. This background meant that time had to be created to generate ideas for interaction and innovation. Innovation tournaments of various descriptors have been proposed and utilised as an idea generator forum (12). Based on this, the Darwinian team (Davis, Lee, Ulrich, Girotra, and Terwiesch) have developed an innovation tournament tool Darwinator (13) which assists in the core tournament processes of idea generation, idea shortlisting, idea pitching and idea selection. This was brought to AccessHC after the Author's participation in an INSEAD Program Innovating Health for Tomorrow (14). The key issues for implementation at AccessHC were selecting the participants, background learning and creating the time for staff to participate. The time pressures were acute as AccessHC was in a constrained funding situation and not all staff were located at the same location. It was decided to limit the tournament to managers and above. A short YouTube video was prepared on the basics of the innovation tournament and an email campaign instituted to solicit initial ideas over a period of several weeks culminating in an innovation workshop. At the day long innovation workshop, the group of about 20 managers generated 125 ideas with a final shortlist of about five decided at the day workshop.

The second iteration of an innovation tournament was an organisation wide innovation tournament with no face-to-face component but with YouTube videos to support the process supported by staff emails. This yielded only 25 ideas and very limited engagement despite email follow ups.

The final iteration was to ask the University to utilise the Darwinator tool. This had limited engagement by the University who indicated that it had its own tools but did not generate any take up by the University.

The key learnings from these exercises is that innovation tournaments require much work to promote engagement particularly in an organisation such as AccessHC with little background in innovation, facing day to day operational challenges. Face to face and dedicated time seems quite fundamental for success and this fits with other studies that participation intensity is required for tournaments to be successful (15).

### Generating the Ideas: The Innovation Pitch

Swinburne University arranged an innovation pitch session. Within the University there was an invitation for academics to consider if their work areas could align with AccessHC. There were preliminary meetings between senior Swinburne and AccessHC staff where the background of AccessHC was explained. These meetings were a combination of understanding AccessHC values and history and needs. This was followed up with a broad outline of intent of the collaborations and hints at potential opportunities. They were facilitated by the Swinburne Office for Partnership and Collaboration and involved Senior Executives of AccessHC. Following up on this was a Swinburne wide invitation for staff to participate in a “pitch session.” The pitches were in a formal context with Swinburne staff pitching to AccessHC executive and management in the audience. No decisions were made at the pitch session which in general were descriptions of current Swinburne Research directions seeking alignment with potential projects at AccessHC. At the end of the sessions, introductions were made between the two groups for further discussion and development which in many cases led to a submission to the Kickstart Program. Those staff unable to pitch were able to make a written submission to the Kickstart Program. From the pitches about 12 opportunities were presented to the Kickstart Program.

### The Kickstart Program: Making Things Happen

The Kickstart Program was funded by AccessHC to stimulate interactions with Swinburne University and was managed by a Kickstart Committee. In the initial iteration the projects emanated from AccessHC and had a design focus: Design of the Consulting room of the future and the Waiting room of the future both were initial projects. In both projects the biggest findings were the feedback from patients and clients consulted by the University staff and students involved rather than the design work itself. These findings continue to influence program design even if laterally. After the 2 years of operation the Kickstart Program was presented with a larger list of potential projects from both AccessHC and Swinburne University where only a few were actually funded. However, in many cases, even the unfunded projects were used to bring academic staff and clinical staff together and the projects outcomes were facilitated without funding allocations. In recent times the projects have been more likely to have a clinical or advocacy dimension. For example, recent projects were on the value of routine exercise on treating depression and the social media influences on alcohol drinking behaviours both of which are in the process of being published. Importantly both may produce changes in AccessHC strategy in its mental health and alcohol and other drug programs. Ultimately, the seed investment by AccessHC opened the doors to wider interaction and positive return on investment. This is the third step of the process in Figure 1.

### One Idea Leads to Another

Whist directed research and innovation was funded in Kickstart, Kickstart was always meant to seed ideas so that they could develop in multiple directions. An example was the waiting room project. Swinburne who has a Design School was encouraged through the Kickstart Program project to design a clinical waiting room of the future. The project was suggested by AccessHC as a response to one of its outdated clinic waiting rooms within a repurposed heritage Post Office which was over 100 years old. This project started with a needs-analysis of needs by Swinburne University over a 6 month period with interviews of patients and staff in the current waiting room. The written report was created to inform the design. However, University staff linked with their internal University animations expertise to better communicate the needs-analysis to AccessHC. The “Access me Not” animation was created, unknown to the staff at AccessHC as a clarity aid to the written report [see (16)]. At initial presentation, the way the animation communicated the patient experience was not envisioned by AccessHC. “Access me not” was submitted for the 2018 International Design Awards and received an honourable mention.

“Access Me Not” is not publicly available as it is an internal document. However, the use of animations to explain a patient experience was new for AccessHC staff. Many of the issues in primary health revolve around patient engagement (17) and the Swinburne animation was a new avenue for AccessHC staff to explore. For example, AccessHC staff saw other uses for the approach and contacted Swinburne to design an animation of a client journey for the newly introduced National Disability Insurance Scheme (NDIS) a new scheme to support members of the community with a disability. The co design produced an animation of immense help to parents of children with developmental difficulties in navigating the scheme for complex and chronic disability care. For AccessHC the scripting was timely and served as a framework to develop it new internal NDIS care systems and processes [see (18)]. The Swinburne team is now producing child health navigation animations for a range of health organisations. Latest iterations include producing the animation in other languages and a Chinese language version has been completed. It is unlikely that in a traditional project funding process that the serendipity effects could be as easily exploited as happened through Kickstart. In the case of Access Me Not, the total ultimate funding for all of the downstream initiatives was probably equivalent to a regular funded project but it came in small quickly developing steps worked on by both AccessHC and Swinburne.

### Traditional Interactions Value-Add

From the very outset, the relationship did not rely on a prescribed activity or research area but on facilitating the relationship. However, there were also traditional interactions which acted as trust building activities. Trust is recognised as a major enabler of Community-University collaborations (19). AccessHC opened it clinical areas to provide placement training for postgraduate Swinburne students in occupational therapy. Indeed, the clinical training is a well-recognised way of Universities seeing benefit in interacting with health services through meeting accreditation requirements in professional degrees, preparing students for the workplace and staying contemporary with current clinical practise (20). This linkage proved valuable for both organisations. Often clinical training places are limited and Universities struggle to deliver the workplace training required for accreditation purposes. The AccessHC environment of community as well as clinical services opened enhanced learning opportunities particularly in interacting with diverse client groups. The benefits were mutual. Swinburne University hosted TOM Makeathon (21), which was a hackathon weekend to develop aids for those with a disability in a multi-disciplinary innovation workshop (22). The initial event, which now happens annually, was hosted by Swinburne University. AccessHC occupational therapy staff were invited to participate. This opened a new innovation opportunity beyond their day-to-day clinical requirements. Staff feedback and engagement were facilitated by the opportunity and an example outcome was a prototype for a portable wheelchair ramp where AccessHC staff participated as part of a multidisciplinary team (23). Without the Swinburne association it would have been unlikely that the event would have engaged AccessHC staff.

### Outcomes

Some of the outcomes from the collaboration are listed on the Swinburne website (24). They include significant health service design tools and work such as a homelessness tool, a social prescribing framework and design work some of which is also published and informs AccessHC practise and service delivery (25–27). The animation work on the AccessHC you tube channel (25) and numerous other publications and conference presentations. The animation work led to a wider use of the format in health information campaigns beyond AccessHC. AccessHC also used private animations custom produced by Swinburne in advocacy campaigns with politicians. These in part may have resulted in wider health policy development.

The more intangible outcomes were participation in strategy development in both organisations. AccessHC had staff represented on Swinburne Advisory Committees such as supporting the foundation of the Swinburne Living Lab Initiative (28) and conversely, Swinburne academic representation on the youth mental health Headspace: Hawthorn service (29) that AccessHC led the formation of. The outcomes are ongoing.

## Discussion

The essence of the case is a planned interaction (Figure 1) between a University (Swinburne) and a small primary health service (AccessHC). The planned interaction replaced previous piecemeal approaches based on individual relationships. Swinburne was used to dealing with large and even multi-national strategic partners. However, there was an alignment of values with AccessHC where impact in the local community was important to the University and embedded in the Swinburne Strategic plan (30). This local community view was explicit in the Swinburne Strategic Plan and gave alignment to the initial relationship with AccessHC. AccessHC had “innovation” as a value (29) but its day to day operational requirements made this somewhat aspirational. Its embeddedness into community was a fundamental part of the AccessHC strategic plan (31). Discussions with AccessHC at the beginning was an exploration of values and the history of AccessHC which is a health charity with a 150 year history (32). Without values alignment it is unclear whether the relationship would have started The groundwork was laid from that shared perspective through multiple background meetings.

Four elements were important in setting the framework for the relationship and making collaboration easy. The first was the Swinburne Office of Collaboration and Partnership who guided the discussions within the University, organised meetings and promoted the potential relationships across all discipline areas within Swinburne. This was a significant resource commitment by the University maintained over 4 years of organisational and personnel change. It maintained momentum throughout.

The second was a formal memorandum of understanding between AccessHC and Swinburne (11) which was a public affirmation of the relationship particularly for staff of both organisations in deciding whether to engage or not. Within AccessHC this also played a role within its Board of Governance highlighting the strategic importance of the activity and elevated the relationship to a major imperative.

The third element, making collaboration easy, was a master research agreement which once negotiated simplified approvals of subsequent projects and removed bureaucracy. It was not unusual for a 24 h approval process.

The final element was the AccessHC Kickstart Program. This was funded entirely but modestly by AccessHC. There have been 5 years of operation and it continues. It served as both a conduit for projects and a beacon for the relationship which everyone could point to. In a transactional sense it was a joint forum for the approval of projects. Without the Kickstart Program there would not have been such a visible sign of potential collaboration. The visibility of Kickstart was important for both organisations in justifying the effort in the relationship.

There were several ways tried to generate projects. The results of innovation tournaments were mixed. An intense innovation tournament campaign which was resource intensive produced better results than an online campaign without face-to-face interactions. This is broadly in line with research into characteristics of successful innovation tournaments (12, 15). The Darwinator program (13) which is an innovation tournament platform was a useful aid. However, without intense engagement activities with staff, the tool was not a significant generator of ideas. To some extent this may reflect the need to create workspace and time in a service delivery organisation, such as AccessHC, for innovation to occur. The importance of dedicated resources to manage innovation tournaments has been previously reported (15). This suggests that for innovation tournaments to be successful in a busy work environment that the resource issue in people, time and place are very important. The case suggests that if this cannot be met that the program may not be of benefit.

The pitch session organised by Swinburne for academics to describe their work and ideas was a safe environment for academics. It offered them an opportunity to describe their research interests to AccessHC staff without necessarily adopting a problem-solving mission for AccessHC problems. The lack of relevance in some cases meant a low level of take up. On the other hand, it proved useful in developing connexions and subsequent discussions between the two staff groups which in some cases resulted in joint projects at a later time. This highlighted that successful projects satisfied both the AccessHC goal and the Swinburne goal.

The missing part of the case study was a robust mechanism for the generation of ideas, problems and solutions which involved both organisations and which would lead to active projects. From the learnings, a well-resourced and facilitated innovation tournament involving both organisations with face to face components seems to offer most prospects. It would fulfil the getting together for a purpose with the added benefit of shared ideas generation.

The role of serendipity or unforeseen consequences cannot be under-estimated in generating both projects and outcomes. The relationship spawned unforeseen projects, events and ideas. The easiest example was the consequences of the design project to create a waiting room of the future. The waiting room design project initiated by AccessHC and fulfilled by Swinburne led to the wide use of animations to help navigate health programs within AccessHC but also beyond to other organisations (33). It was borne by Swinburne colleagues using animations to describe their analysis of waiting room issues to AccessHC and was completely unexpected by AccessHC. The reason for the success of serendipity probably rested with the Kickstart funding being small, flexible and easy to approve. This meant that there was little risk in halting or changing directions. Some of those direction changes were funded independently of Kickstart fulfilling the seed funding ambition of Kickstart.

### Influence of COVID

In 2020 Australia had stringent international and interstate travel bans due to COVID-19. Melbourne had a prolonged hard lockdown of 112 days (34). This affected both organisations in different ways. For Swinburne University, the most of 2020 was without on-campus learning and most staff were working from home. For AccessHC COVID was a major health event to which it had to respond with staff having to deliver more health and community services safely in very constrained circumstance. Even in the midst of the COVID waves, the two organisations discussed how they could work together in setting up boutique manufacturing of face masks through the Swinburne Manufacturing faculty and repurposing of laboratory supplies to functional hand sanitizers. Neither eventuated, but the supportive relationship continued.

The Swinburne project was put on hold during COVID. However, it seems resilient and coming out of COVID in 2021 both organisations have re-committed to the relationship. The formal Memorandum of Understanding was updated for a new signing with a new University Vice Chancellor and new CEO of AccessHC. The Kickstart projects previously not allowed to operate during lockdown in 2020 are again in operation. The Kickstart Committee reviewed it operations to again solicit projects and ideas later in 2021.

### The Future

Not all discussion led to viable projects. In some cases, it was because of lack of relevance to AccessHC, lack of interest to University Academics or practical or resource issues. Nevertheless, the relationship endured and continues to generate new projects. The Kickstart Program continues to be a focus to harness projects and ideas. As outlined by Swinburne (19), prior to COVID the majority of “live” projects did not require funding and simply the bringing together of staff of both organisations. The question is whether the case study could be used as a framework to other organisations. The elements appear sound but a missing part of the approach is the unwritten a dedication from both organisations to make it work. Without this upfront commitment the results may not be so evident. Some of the case study elements are instructive in generating commitment and engagement.

## Data Availability Statement

The original contributions generated for the study are included in the article/supplementary material, further inquiries can be directed to the corresponding author.

## Author's Note

Towards the end of the case study, the author was appointed as an Adjunct Professor of Swinburne University of Technology, which is an unpaid honorary position, and served in an unpaid honorary capacity on external advisory committees for the Bachelor Health Sciences at the University and the Swinburne Centre for Mental Health.

## Author Contributions

The author confirms being the sole contributor of this work and has approved it for publication.

## Conflict of Interest

The author was CEO of AccessHC during the case period reported, a paid position with chief executive responsibility.

## Publisher's Note

All claims expressed in this article are solely those of the authors and do not necessarily represent those of their affiliated organizations, or those of the publisher, the editors and the reviewers. Any product that may be evaluated in this article, or claim that may be made by its manufacturer, is not guaranteed or endorsed by the publisher.

## Footnotes

^1^https://scholar.google.com/citations?vie18w_op=top_venues&hl=en&vq=med_primaryhealthcare (accessed 5 July, 2021).
